# Unconscious information processing in executive control

**DOI:** 10.3389/fnhum.2013.00021

**Published:** 2013-01-31

**Authors:** Nicola De Pisapia

**Affiliations:** Department of Cognitive Science and Education, University of TrentoRovereto, Italy

This Frontiers Research Topic includes original experimental studies and reviews on unconscious processing in executive control. Executive control refers to the ability of the human brain—mostly associated with prefrontal cortex activity—to regulate the execution of novel or complex goal-directed tasks. Previous studies and models of human cognition have assumed that executive control necessarily requires conscious processing of information. This perspective is in line with common sense and personal introspection, which suggest that our choices and action regulations are intentional and based on conscious stimuli.

Nevertheless, in the last few years several behavioral and cognitive neuroscience studies have put under scrutiny this assumption. Cumulating evidence is now showing that executive control can involve or be triggered by unconscious processing of information, with consequent effects on observed behaviors. One of the main methods adopted to study such unconscious mechanisms is masked priming, consisting in presenting visually masked stimuli, which nonetheless are shown to affect goal-directed behavior or influence constructs linked to executive control and prefrontal cortex activity (e.g., task-set representation, response inhibition, conflict monitoring, error detection, reward processing, emotion regulation, and task switching). Other methods have been adopted, such as auditory masking or implicit regularities in stimulus presentations.

This area of research is relatively young, complex, and challenging, but very exciting. While scientific evidence is emerging—no general consensus has been reached yet on how to interpret these early findings. Some researchers accept that executive control can involve unconscious processing, others momentarily put aside—in first approximation—this issue, and others criticize this possibility on theoretical grounds (e.g., pointing to the need of better definitions of terms such as control, conflict, and consciousness) or based on experimental findings.

This Frontiers Research Topic is born from the idea that it is time that researchers in cognition make a collective effort to deepen the understanding of the unconscious mechanisms involved in executive control. The book includes articles from top researchers in the field, and it is organized in a first section with a selection of original research articles, and a second section with review and hypothesis papers.

## Section I: original research articles

In the first article of the experimental section, Fuchs and Ansorge (2012) address the question of whether inhibition has the same characteristics in conscious and unconscious executive control. In particular, they focus on the so-called Inhibition of Return (i.e., slower responses to attended than unattended positions), which in previous studies was considered to indicate an automatic capture of visual attention driven by unconscious cues. In a series of experiments, the authors find that Inhibition of Return can only be obtained with conscious cues, thus suggesting that consciousness might be a necessary condition for inhibition.

In the next research article, Gaillard et al. (2012) investigate the relationship between inner speech and consciousness in executive control, and they find that articulatory suppression specifically impairs exclusion performance by interfering with inner speech.

In the next article, Schlaghecken et al. (2012) use a masked prime task to test the hypothesis that typical aging is associated with a selective deficit in inhibitory function, affecting both low-level motor, and higher-level executive control.

Finally for this section, Zedelius et al. (2012) argue in favor of a unique role of consciousness in efficient allocation of effort during cognitive control processes, and they investigate the delivery of unconscious rewards during task executions. They find that people fail to integrate unconsciously perceived reward value with conscious expectancies concerning the attainability of rewards, which leads them to poor and unsubstantiated choices.

## Section II: review and hypothesis articles

Moving on to the next section, McBride et al. (2012) discuss findings concerning object affordances, alien limb syndrome, the visual grasp reflex, subliminal priming, and subliminal triggering of attentional orienting. Their suggestion is that automatic motor activation might form an intrinsic part of all behavior, rather than being categorically different from conscious actions.

Desender and Van den Bussche (2012) critically review the topic of adaptation to response conflict, which is a key component of executive control processes, and they discuss how it can be induced by unconscious information processing.

Kiefer (2012) provides an overview of the latest research on executive control influences on unconscious information processing. He discusses his attentional sensitization model of unconscious information processing, in which unconscious processing is only elicited if the cognitive system is configured accordingly, namely it depends on attentional amplification of task-congruent processing pathways as a function of task-sets.

Meiran et al. (2012) review and discuss the seemingly paradoxical loss of control associated with states of high readiness to execute a plan, termed “intention-based reflexivity.” They suggest that the neurocognitive systems involved in the preparation of novel plans are different than those involved in preparation of practiced plans, and discuss the idea that proactive control (acting in advance of the experienced response conflict) tends to be a relatively rigid construct.

Horga and Maia (2012) suggest that conscious and unconscious processes might be implemented by the same neural substrates and largely perform the same neural computations. What characterizes these processes is time, namely more durable neuronal firing for conscious processes, and less durable neuronal firing for unconscious processes, but both of them can cause behavior.

In the final article, Prabhakaran and Gray (2012) expand the idea of unconscious information processing in executive control by exploring and reviewing the societal unconscious influences, such as priming of goals, social hierarchies, and interpersonal interactions.

This collection of papers offers an exciting overview of the field of unconscious processing in executive control, and hopefully it will help other researchers of cognition to further explore this young and exciting field.
